# Stress-induced despair behavior develops independently of the Ahr-RORγt axis in CD4 + cells

**DOI:** 10.1038/s41598-022-12464-2

**Published:** 2022-05-21

**Authors:** Courtney R. Rivet-Noor, Andrea R. Merchak, Sihan Li, Rebecca M. Beiter, Sangwoo Lee, Jalon Aaron Thomas, Anthony Fernández-Castañeda, Jung-Bum Shin, Alban Gaultier

**Affiliations:** 1 Center for Brain Immunology and Glia, University of Virginia School of Medicine, Charlottesville, VA 22908 USA; 2 Department of Neuroscience, University of Virginia School of Medicine, Charlottesville, VA 22908 USA; 3 Graduate Program in Neuroscience, University of Virginia School of Medicine, Charlottesville, VA 22908 USA; 4 Graduate Program in Biochemistry and Molecular Genetics, University of Virginia School of Medicine, Charlottesville, VA 22908 USA; 5 Undergraduate Department of Computer Science, University of Virginia School of Engineering and Applied Science, Charlottesville, VA 22904 USA

**Keywords:** Psychology, Immunology, Adaptive immunity, Neuroimmunology

## Abstract

Current treatments for major depressive disorder are limited to neuropharmacological approaches and are ineffective for large numbers of patients. Recently, alternative means have been explored to understand the etiology of depression. Specifically, changes in the microbiome and immune system have been observed in both clinical settings and in mouse models. As such, microbial supplements and probiotics have become a target for potential therapeutics. A current hypothesis for the mechanism of action of these supplements is via the aryl hydrocarbon receptor’s (Ahr) modulation of the T helper 17 cell (Th17) and T regulatory cell axis. As inflammatory RORγt + CD4 + Th17 T cells and their primary cytokine IL-17 have been implicated in the development of stress-induced depression, the connection between stress, the Ahr, Th17s and depression remains critical to understanding mood disorders. Here, we utilize genetic knockouts to examine the role of the microbial sensor Ahr in the development of stressinduced despair behavior. We observe an Ahr-independent increase in gut-associated Th17s in stressed mice, indicating that the Ahr is not responsible for this communication. Further, we utilized a CD4-specific RAR Related Orphan Receptor C (*Rorc*) knockout line to disrupt the production of Th17s. Mice lacking *Rorc*-produced IL-17 did not show any differences in behavior before or after stress when compared to controls. Finally, we utilize an unsupervised machine learning system to examine minute differences in behavior that could not be observed by traditional behavioral assays. Our data demonstrate that neither CD4 specific *Ahr* nor *Rorc* are necessary for the development of stress-induced anxiety- or depressive-like behaviors. These data suggest that research approaches should focus on other sources or sites of IL-17 production in stress-induced depression.

## Introduction

In the United States, an estimated 17.3 million adults are diagnosed with major depressive disorder (MDD). With over 60% struggling with severe impairments, MDD is the number one cause of disability in the U.S.^1^. Dogma states that depression and other mood disorders are caused by an imbalance in neurotransmitters^2–4^. As such, the majority of existing treatments target neurotransmitter uptake (SSRIs, SNRIs, etc.)^5^. However, a significant number of patients do not benefit from these therapeutics, suggesting alternate etiologies for MDD^5^.

The microbiome has emerged as an important contributor to many neurological conditions, ranging from Parkinson’s disease to autism spectrum disorder^6,7^. Thus, it is unsurprising that the microbiome has also been found to play a crucial role in the development and maintenance of MDD. For example, patients with depression have altered microbiomes when compared to control patients^8^. Microbiome dysbiosis is also present in animal models of MDD^9–12^ and therapeutic administration of probiotics has been found to be beneficial^12^. However, the mechanism by which the microbiome contributes to the etiology of depression remains under investigation. One postulated route is through the immune system^13^.

While there are many ways in which inflammation and the microbiome can interact in depression, a microbiome-responsive regulator of the immune system called the aryl hydrocarbon receptor (Ahr) has the potential to serve as a lynchpin in this pathway. Indeed, metabolomics, GWAS, and epigenetic studies have indicated that dysfunction of the Ahr is associated with both MDD and post traumatic stress disorder^14,15^. The Ahr is a cytoplasmic receptor that can bind microbiome produced metabolites, including tryptophan-derived molecules^13^. Ahr activation induces transcription factor activity that can modulate the inflammatory environment of the gut. In particular, the Ahr has been shown to regulate T helper cell 17 (Th17) and T regulatory cell (Treg) function in response to microbial metabolites^16^. This represents an important connection between the microbiome and immune system as IL-17 produced by ROR$\upgamma$t + Th17s have been shown to contribute to depression- and anxiety-like behaviors^17,18^. Studies have shown that the number of Th17s in the gut associated lymphoid tissue (GALT) increases in response to stress and correlates with depressive-like behavior^19^. Additionally, the transfer of Th17 cells into mice has been found to induce depressive-like behaviors^17^. Administration of IL-17A blocking antibodies has also been found to reduce learned helplessness behaviors in mice^17^. However, the evidence for the role of IL-17 in MDD remains controversial, with some reports claiming a positive correlation between the inflammatory cytokine and depression and others reporting no differences in IL-17 levels between those with MDD and controls^20–25^. These conflicting results highlight the need for further investigation into the mechanism and role of the Ahr and increased inflammatory Th17 cells in depression.

Here, for the first time, we use genetic tools and artificial intelligence driven behavior analysis to assess the contribution of T cell specific Ahr and RORγt to the development of anxiety- and depressive-like behaviors in a murine model of stress. While our data confim that stressed mice present with a larger number of Th17 cells in the gut, our behavioral analyses show that the deletion of either *Ahr* or *Rorc*$\upgamma$ in T cells does not impact anxiety- or depressive-like behaviors in mice. Taken together our data suggest that ROR$\upgamma$t induced IL-17 in T cells is not necessary for the pathological development of depressive-like behaviors induced by unpredicalble chronic restraint stress.

## Results

### Absence of the Ahr in T cells does not influence baseline behaviors

**Figure 1:**
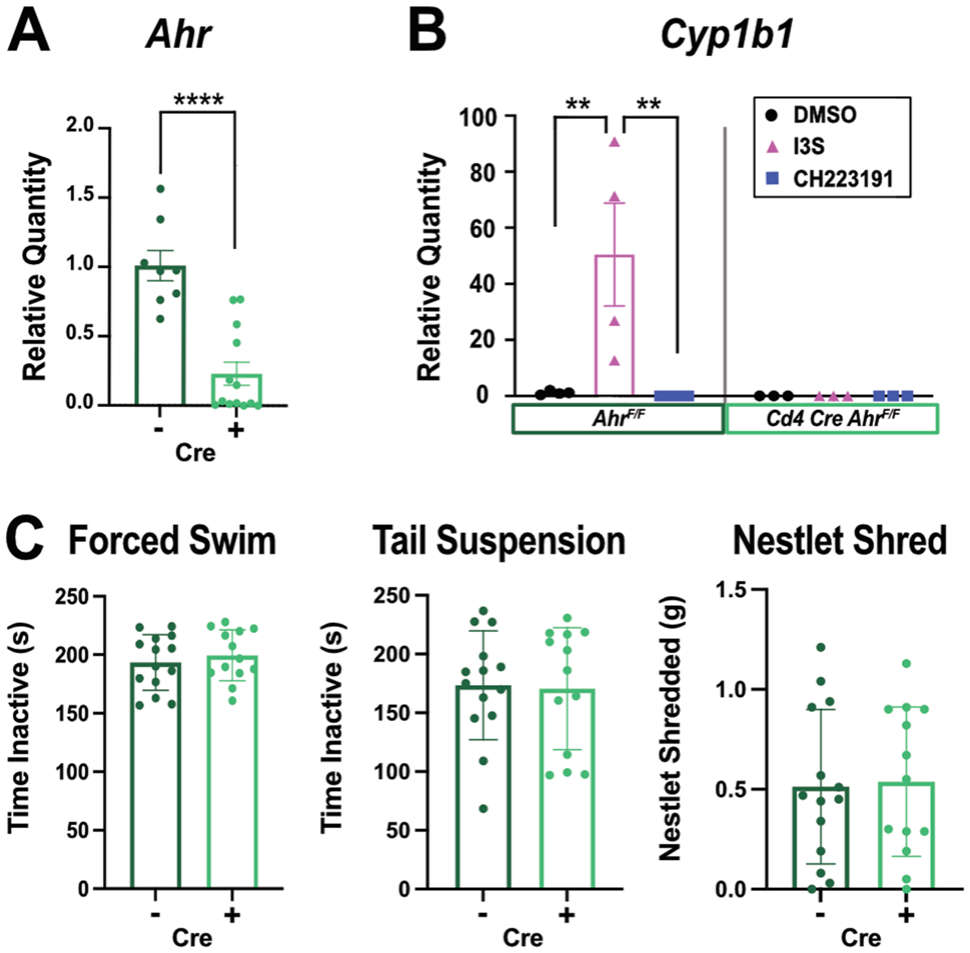
Absence of *Ahr* in T cells does not Influence Baseline Behavior. (**A**) Relative quantity of *Ahr* expression from in vitro skewed Th17 cells in *Ahr* KO and *Ahr* competent cells was determined by qPCR (n = 8 or 13/group). T test (*p* =  < 0.0001). (**B**) Relative quantity of *Cyp1b1* expression from *Ahr* KO and competent cells skewed in vitro to become Th17s and treated with an Ahr agonist (I3S), antagonist (CH223191), or control media (DMSO) (n = 3–4/group). Combined N = 2. Two-way ANOVA (DMSO to I3S: *p* = 0.0019; I3s to CH223191: *p* = 0.0016, interaction statistics in Supplemental Table 1). (**C**) Baseline behavioral comparisons between *Ahr* KO and littermate controls (n = 13–14/group). T tests. Male mice.

We and others have previously shown that microbiome dysbiosis induced by unpredictable chronic stress (UCS) is a contributing factor to depressive- and anxiety-like behaviors in mice^12,26,27^. Furthermore, therapeutic reconstitution with *Lactobacillus* is sufficient to correct behaviors induced by UCS^12^. *Lactobacillus* is known to produce metabolites that engage the Ahr receptor, such as tryptophan metabolites which can modify the immune environment^28^. Given the emerging role of T cells and their cytokines in depression^17,29,30^, we wanted to determine whether T cell-specific Ahr sensing of the microbiome leads to the immunological and behavioral changes associated with stress. We generated *Cd4 Cre Ahr*^flox/flox^ mice (*Ahr* KO) to examine depressive- and anxiety-like behaviors in the absence of this important receptor in the T cell compartment. As expected, *Ahr* expression was significantly reduced in CD4 + T cells isolated from *Ahr* KO mice by qPCR (Fig. 1A). Furthermore, in vitro generated Th17 cells prepared from *Ahr* KO animals failed to upregulate the Ahr downstream transcription factor *Cyp1b1* in response to idoxyl-3-sulfate (I3S) induced Ahr activation^31^. *Ahr* KO levels of *Cyp1b1* expression were consistent with those of Ahr competent cells treated with an Ahr antagonist CH223191. This confirmed a robust functional deletion of Ahr in T cells (Fig. 1B). Next, we explored whether lacking Ahr in T cells could influence behaviors at baseline. Both depressive- and anxiety-like behavioral assays were performed on *Ahr* KO mice and age-matched controls. No differences were observed in the forced swim, tail suspension, or nestlet shredding tests (Fig. 1C). These results show that mice lacking Ahr in T cells do not present with baseline depressive- or anxiety-like behaviors.

### UCS drives a specific expansion of Th17 cells in the lamina propria in an Ahr-independent manner

**Figure 2:**
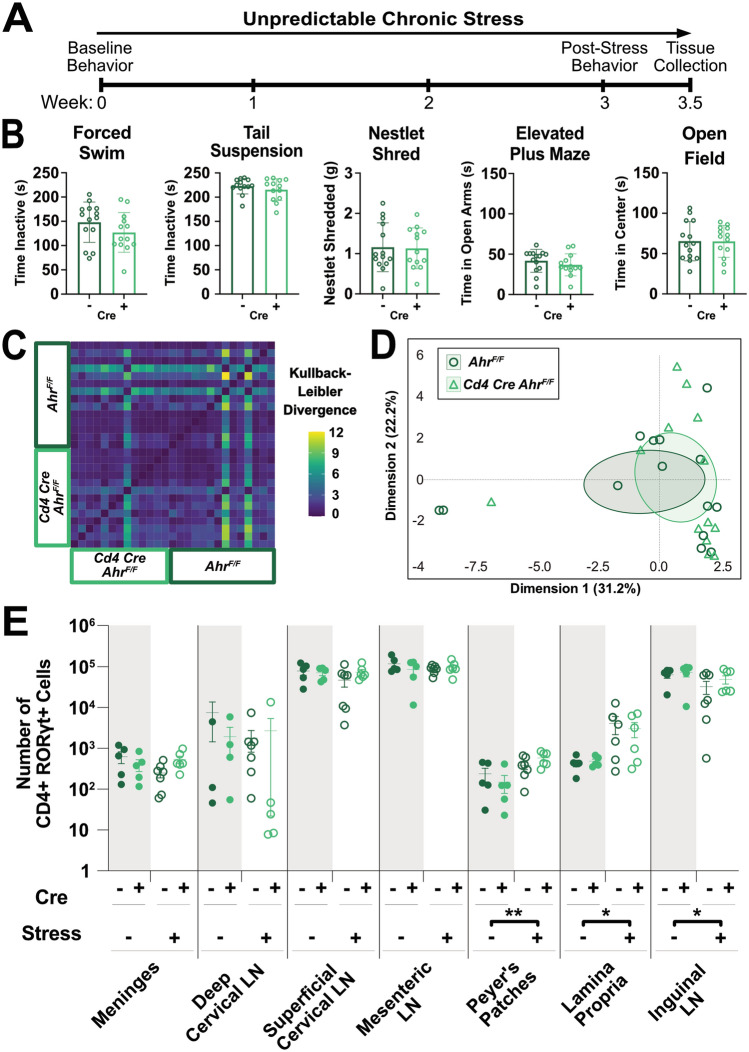
Unpredictable Chronic Stress (UCS) Drives Expansion of Th17s in the Lamina Propria in an *Ahr* Independent Manner. (**A**) Schematic representing timeline for UCS in mice. (**B**) Measures of learned helplessness and anxiety-like behaviors between *Ahr* KO and littermate controls (n = 13–14/group). T tests, male mice. (**C**) Kullback–Leibler divergence heatmap and (**D**) PCA plot of DeepLabCut analyzed behaviors between groups (n = 13–14/group). A single representative experiment is shown (N = 2). (**E**) Number of CD4 + ROR$\gamma$t + cells in various immune tissues between stressed and naïve *Ahr* KO and littermate controls (n = 5–7/group). Two-way ANOVA (Peyer’s Patches: *p* = 0.0026, Lamina Propria: *p* = 0.0359, Inguinal LN: *p* = 0.0440), N = 1, male mice. LN = lymphnode.

To test the role of the T cell Ahr in response to stress, we subjected *Ahr* KO or age-matched controls to 3 weeks of UCS, as previously described^12^ (Fig. 2A and Supplemental Table 2). Surprisingly, no differences in anxiety- or depressive-like behaviors were observed between groups after stress (Fig. 2B). While classic behavioral assays may detect strong phenotypic differences, they may not detect more subtle behavioral changes. To circumvent this limitation, we applied an unsupervised machine learning approach to analyze behaviors in both genotypes using DeepLabCut^32^. First, we validated this computational approach using a preclinical murine model of multiple sclerosis (MS) known as experimental autoimmune encephalomyelitis (EAE). EAE produces significant locomotor changes and should produce detectable motor differences between groups to act as a positive control. DeepLabCut was able to detect robust behavioral differences between groups in nearly 40% of behavioral motifs characterized by the software (Sup Fig. 1A–F). However, in our *Ahr* KO vs control groups, Kullback–Leibler Divergence, a measure quantifying variance within and between groups (Fig. 2C and Sup Fig. 1B), showed more variance within a group than between groups, suggesting that individual mice have a stronger impact on behavior than the genotype. Similarly, the generated PCA plot showed large overlap between groups (Fig. 2D). Of all behavioral motifs analyzed, only one showed a significant change between groups (2.8% of all behaviors), suggesting the overall behaviors of *Ahr* KO mice are very similar to control animals in response to stress (Sup Fig. 2C,D). Finally, immunophenotyping revealed no differences in the measured immune compartments between *Ahr* KO and control mice (Sup Fig. 2A). Interestingly, both the *Ahr* KO animals and controls showed an increase in the number of CD4 + ROR$\upgamma$t + Th17 cells in the GALT that was significantly impacted by stress (Fig. 2E). We also observed a genotype independent decrease in Th17s in the inguinal lymphnodes. We believe this is due to cells migrating out of the lymphnodes and into the GALT. Together, these data suggest that the microbiome changes and increases in Th17 cells observed in response to stress are not mechanistically linked by Ahr activation in CD4 + cells.

### Deletion of *Rorc* in T cells does not induce spontaneous anxiety- or depressive-like behaviors

**Figure 3:**
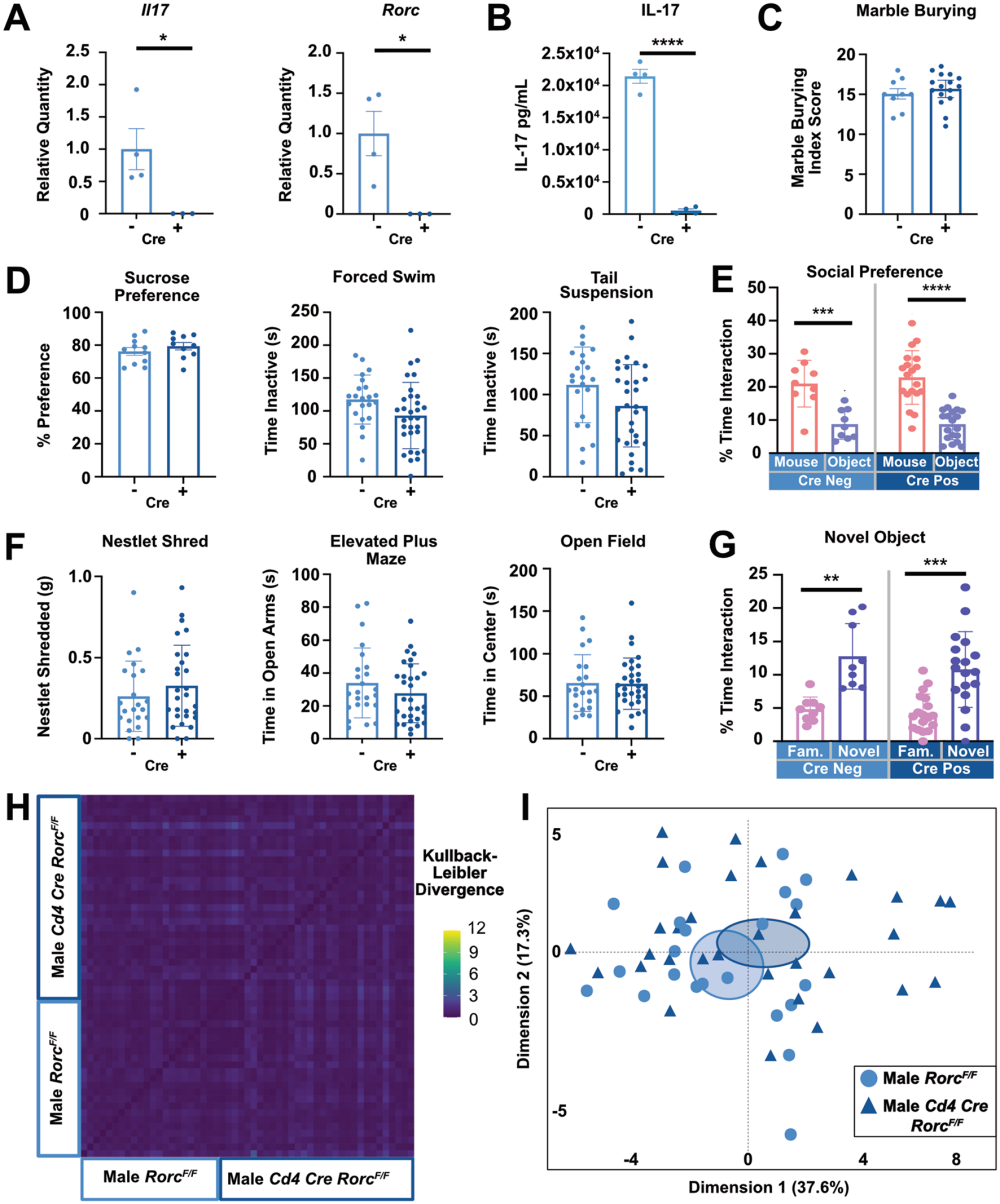
Depletion of *Rorc* does not Induce Spontaneous Behavioral Changes in Male Mice. (**A**) Loss of gene expression of both *Il17* and *Rorc* in in vitro skewed Th17s from *Rorc* KO mice and littermate controls by qPCR (n = 3–4/group). T tests (*il17*: *p* = 0.0450, *Rorc*: *p* = 0.0281). (**B**) ELISA representing loss of IL-17 in in vitro skewed Th17s from *Rorc* KO animals (n = 3–4/group). T test (*p* =  < 0.0001). (**C**) Baseline marble burying (n = 9 or 16/group), (**D**) sucrose preference (n = 9/group) and forced swim and tail suspension (n = 22 or 37/group), (**E**) social preference (n = 9 or 19/group), (**F**) nestlet shredding (n = 22 or 28/group), elevated plus maze (n = 24 or 31/group) and open field (n = 22 or 31/group), and (**G**) novel object recognition (n = 9 or 19/group) tests. T tests used in D and F. Two-way ANOVA used in E (Cre Neg: *p* = 0.0010, Cre Pos: *p* < 0.0001) and G (Cre Neg: *p* = 0.0014, Cre Pos: *p* = 0.0002). (**H**) Kullback–Leibler divergence heatmap and (I) PCA plot of DeepLabCut analyzed behaviors between groups (n = 22 or 32/group). Combined N = 2, male mice.

To explore the role Th17 cells in anxiety- and depressive-like behaviors, *Cd4 Cre Rorc*^*flox/flox*^ (*Rorc* KO) mice were generated. Knock down of ROR$\upgamma$t was confirmed using in vitro-derived Th17s by examining levels of *Il17* and RAR Related Orphan Receptor (*Rorc)* by qPCR (Fig. 3A) and IL-17 secretion by ELISA quantification (Fig. 3B). Immunophenotyping on the spleen revealed the absence of CD4 + ROR$\upgamma$t + T cells in vivo (Sup. Fig. 3A–C). Baseline behaviors were compared between male *Rorc* KO mice and controls. No differences between the *Rorc* KO mice or age matched controls were observed in tasks used to assess depressive- (Fig. 3D) or anxiety- like behaviors (Fig. 3F). As Th17 cells are also known to contribute to autism-like behaviors in male mice^18^, we analyzed the marble burying (Fig. 3C), social preference (Fig. 3E) and novel object recognition (Fig. 3G) tests at baseline. No differences between genotypes were observed in these behavior tests. Lastly, to ensure there were no subtle behavior differences between groups that could not be quantified with known behavioral tasks, we again used machine learning to examine unbiased behavioral clusters. The Kullback–Leibler Divergence plot showed individual variance had a larger impact on behavior than genotype effects (Fig. 3H and Sup Fig. 3D) and a large overlap in the PCA plot was observed (Fig. 3I). Additionally, only 3 of 35 behavioral motifs demonstrated significant differences between groups after DeepLabCut analysis (Sup. Fig. 3C–F). Together, these data suggest that at baseline, a lack of *Rorc* from development does not impact autism-, depression-, or anxiety-like behaviors in male mice.

### UCRS induced anxiety- or depressive-like behaviors are not affected by the lack of *Rorc* in Th17 cells

**Figure 4:**
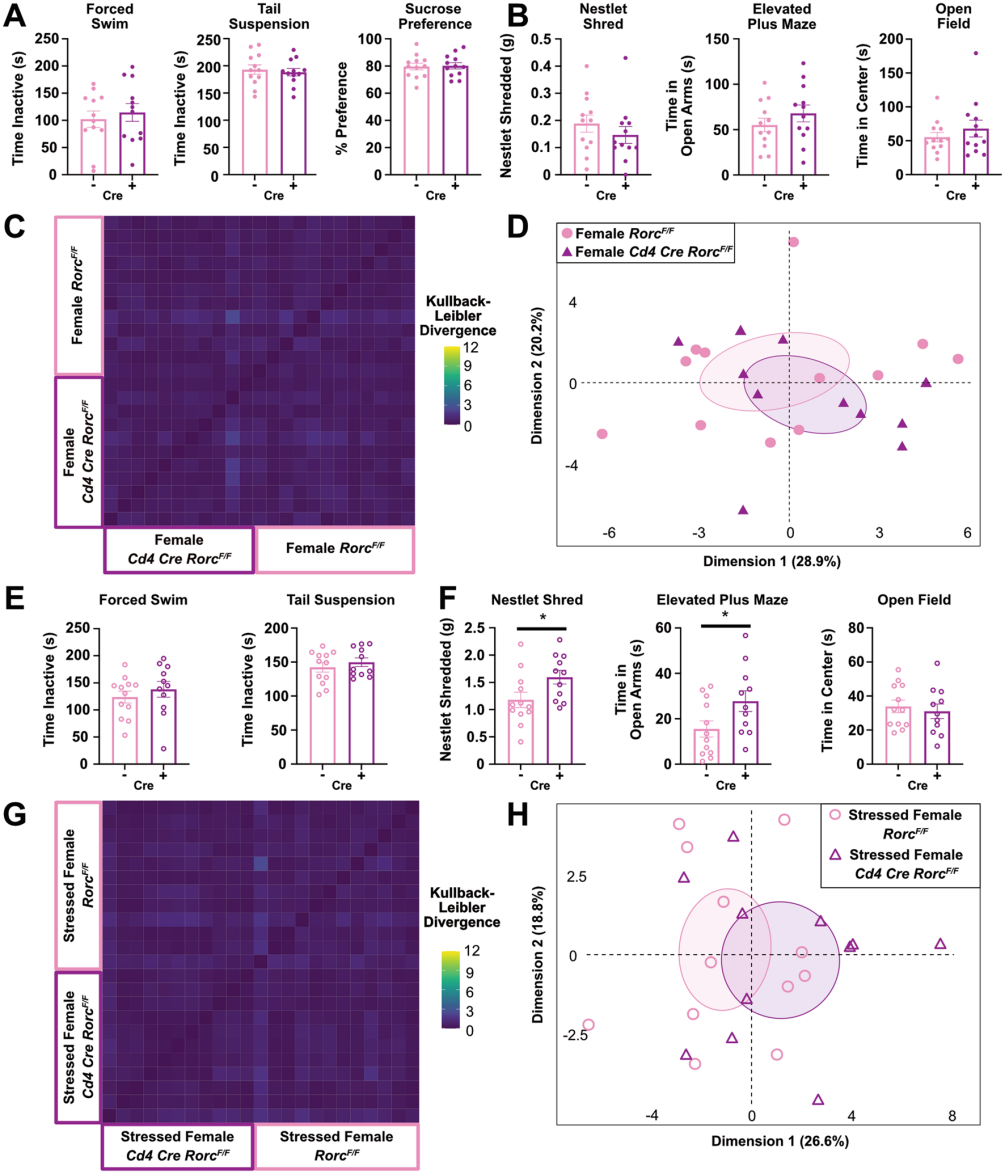
Loss of *Rorc* in T cells does not Impact Depressive- or Anxiety- like Behaviors in Female Mice. No differences in (**A**) depressive-like (n = 12/group) or (**B**) anxiety-like (n = 12/group) behaviors between female *Rorc* KO and littermate controls at baseline. No differences in the (**C**) Kullback–Leibler divergence heatmap or (**D**) PCA plot of DeepLabCut analyzed baseline behaviors between groups (n = 12/group). (**E**) Depressive-like (n = 12/group) and (**F**) anxiety-like (n = 12/group) behaviors after 3 weeks of UCRS between female *Rorc* KO and littermate controls (Nestlet Shred: *p* = 0.0392, Elevated Plus Maze: *p* = 0.0453). (**G**) Kullback–Leibler divergence heatmap and (**H**) PCA plot of DeepLabCut analyzed behaviors after 3 weeks of UCRS between groups (n = 12/group). T tests, N = 1, female mice.

While no behavioral differences in male mice were observed between groups at baseline, females experience depression at a higher rate than males^33^. Thus, we aimed to examine the impact of *Rorc* in Th17 cells in female mice. No differences in escape behavior, anhedonia, or anxiety-like behaviors were observed between female *Rorc* KO mice and controls at baseline (Fig. 4A,B). Similarly, our machine learning approach was not able to detect differences in behaviors as shown through the Kullback–Leibler Divergence plot (Fig. 4C and Sup. Fig. 4C), PCA plot (Fig. 4D), and motif usage breakdown plots (Sup. Fig. 4A,B). To examine the impacts of *Rorc* KO in a stressful environment, female mice were exposed to 3 weeks of Unpredictable Chronic Restraint Stress (UCRS), a stronger model of stress known to induce anxiety- and depressive-like behaviors and changes in spine density of the basolateral amygdala^34^. After exposure to stress, no differences between groups were observed in escape behavior (Fig. 4E). The nestlet shred test revealed that *Rorc* KO mice demonstrate a significant increase in anxiety-like nesting behaviors, whereas the elevated plus maze showed a significant decrease in anxiety-like behaviors, and the open field test showed no difference between groups (Fig. 4F). Ultimately, our unbiased computational approach also did not detect any genotype driven differences as seen in the Kullback–Leibler Divergence plot (Fig. 4G and Sup. Fig. 4F) and the PCA plot (Fig. 4H). No differences in groups were detected in any of the 35 behavior motifs identified between groups. In summary, although we saw a significant increase in anxiety-like behavior in the nestlet shredding test between *Rorc* KO and control animals, our other behavioral tests did not support this trend and our unbiased behavioral analysis showed no changes between groups. Based on these data, *Rorc* KO in T cells does not significantly impact anxiety- and depressive-like behaviors in female mice before or after UCRS.

## Discussion

Depression presents a major social, health, and economic concern across the world, representing the most common cause of disability^35^. The root causes of this debilitating disorder have yet to be elucidated. With treatments remaining inconsistent and new options lacking, new research is needed to further our understanding of this disorder. Both the microbiome and T lymphocytes have been gaining support as potential mediators of MDD pathology. Here, we examined the role of T cell specific ROR$\upgamma$t and Ahr in a mouse model of depression. We demonstrate that deletion of the *Ahr* gene does not impact anxiety- or depressive-like behaviors at baseline or after chronic stress exposure. Additionally, while we see an increase in the number of ROR$\upgamma$t + cells in the lamina propria of stressed mice, it occurs independently of the Ahr. This indicates that there is another mechanism for Th17 cell expansion in response to stress other than environmental sensing through the Ahr.

Further, when *Rorc* is deleted from CD4 + T cells, no changes in anxiety or depressive-like behaviors are observed at baseline or after stress exposure. This is supported by previous work showing that increasing Th17s is not sufficient to change the susceptibility of mice to social defeat stress^29^. Others have suggested that increased Th17s are responsible for stress-induced depressive-like behaviors and have supported this claim by transferring Th17s into mice and finding increases in depressive-like behaviors^17^. However, this only suggests that an increase in Th17s may be sufficient to drive these behaviors^17^, not that it is necessary. Additionally, while Th17’s signature cytokine IL-17 is implicated in mood disorders, the origin of IL-17 is not precisely defined. IL-17 can be produced from many cell types and be induced in alternative ways that may contribute to depression outside of Th17s^36,37^. Supporting this concept, it has been found that IL-17 from $\mathrm{\gamma \delta }$ T cells regulates anxiety-like behaviors in mice^30^. Additionally, multiple transcription factors (STAT3, NF-$\mathrm{\kappa B}$, KLF4, etc.) and microRNA can act on IL-17 producing cells to induce IL-17 production^38^. This regulation can act in synergy with ROR$\upgamma$t or independently of it, as with KLF4^38^, suggesting that IL-17 could be produced without *Rorc* and outside of Th17 cells. These data and our own work suggest that if there is a role of IL-17 in the onset of depression, this cytokine does not solely originate from CD4 + cells or is produced by alternative cell type (ILC3s, $\mathrm{\gamma \delta }$ T cells, or others).

The notion that Th17 cells play a critical role in the onset of depression has been growing in popularity but remains controversial. Several human studies have demonstrated that changes in IL-17 are correlated with depression^21,23,25,39^ while others have not^20,24^. While further work is needed to fully understand the role of IL-17 in the onset of depressive symptoms, our work demonstrates that the Ahr is not responsible for the observed stress-induced increase in intestinal Th17s and that these cells are not necessary for the induction of stress-induced depression. Instead, we suggest there are alternative means or sites of IL-17 production in stress-induced depression. Further investigation of the mechanisms and potential causes of depression symptoms, including the microbiome and other inflammatory signals, is required.

## Materials and methods

All methods were performed in accordance with guidelines and regulations of the University of Virginia and approved by the University of Virginia Animal Care and Use Committee.

### Mice

B6.Cg-Tg(Cd4-cre)1Cwi/BfluJ (CD4Cre)^40^ (#022071), B6(Cg)-*Rorc*^*tm3Litt*^/J (Roryt^fl^)^18^ (#008771), and *AHR*^*tm3.1Bra*^/J (Ahr^fx^) (#006203)^41^ mice were purchased from Jackson Laboratories. Mice were bred in-house. Mice were kept on a 12-h light/dark schedule. All behavioral interventions were performed between 8 am and 3 pm and animals were sacrificed between 7 am and 1 pm. All mice were housed in cages of up to 5 animals from birth until initiation of the stress protocol. All mice exposed to stress were at least 8 weeks of age and age matched to control animals. Stressed mice were housed individually without enrichment to enhance stress^42^. Naïve animals were housed in standard cages in groups of 2–5 mice of the same sex. All procedures were approved by the University of Virginia ACUC (protocol #3918). All experiments were conducted and reported according to ARRIVE guidelines (https://arriveguidelines.org/arrive-guidelines).

### Experimental autoimmune encephalomyelitis

EAE was induced in 6–8-week old female *Cd4 Cre Ahr*^*F/F*^ and *Ahr*^*F/F*^ mice as previously described^43^. Briefly, mice were subcutaneously injected with MOG 35-55 and Complete Freund’s Adjuvant. Two intraperitoneal injections of pertussis toxin were administered on days 0 and 1. Thirty-minute videos of mice were taken at day 25 post immunization and used for analysis. Healthy controls were age-matched females.

### Stress experiments

In our model of Unpredictable Chronic Stress (UCS), mice were exposed to a 2 h period of a daily stressor (restraint, strobe light, or white noise). After the daily stressor, mice were placed in an overnight stress (cage tilt, 24 h light exposure, wet bedding, or 2 × cage change) until the next induction of 2 h stress (Supplemental Table 2). UCS protocols were maintained for 3 weeks. For Unpredictable Chronic Restraint Stress (UCRS) experiments, mice were exposed to chronic restraint (ventilated 50 mL conical vials) for a period of 2 h daily for a period of three weeks. Once removed from restraint, an overnight stressor of either cage tilt, wet bedding, or 2 × cage change was used (Supplemental Table 2). All daily stressors were carried out between 8 am and 5 pm. Overnight stressors were started upon removal from the daily stressor and remained in place until the next day’s daily stressor.

### Behavioral tests

The forced swim, tail suspension, sucrose preference, open field, elevated plus maze, novel object recognition, marble burying, and three chamber social preference tests were performed as previously described^7,44–50^. All testing was recorded on a Hero Session 5 GoPro and analyzed with Noldus behavioral analysis software.

### DeepLabCut

*Animal pose estimation:* Animal pose estimation was performed by using a deep-learning package, DeepLabCut^32^ (https://github.com/DeepLabCut/DeepLabCut). We generated a DeepLabCut convolutional neural network to analyze open field test videos, which is trained in a supervised manner: 16 manually labeled points were selected as references of transfer learning. 15 randomly selected videos were used for building a training dataset. Finally, the performance of the neural network is evaluated by researchers.

*Unsupervised behavior classification*: Estimated mouse poses from DeepLabCut were further analyzed by Variational Animal Motion Embedding (VAME)^51^, which classifies animal behavior in an unsupervised manner (https://github.com/LINCellularNeuroscience/VAME). We trained a unique VAME recursive neural network for each experiment, which classifies each frame of the open field test video into 1 of the 35 behavioral motifs. Then, all behavior motifs were annotated and evaluated by blinded researchers. With annotated frames, we were able to calculate the percentage of time usage of each motif, which is then used for principal component analysis and Kullback–Leibler divergence analysis.

### RNA extraction and quantitative PCR

For RNA extraction, cultured cells were pelleted, frozen, and lysed. RNA was extracted using the Bioline Isolate II RNA mini kit as per manufacture’s protocol (BIO-52073). RNA was quantified with a Biotek Epoch Microplate Spectrophotometer. Normalized RNA was reverse transcribed to cDNA with either the Bioline SensiFast cDNA Synthesis Kit (BIO-65054) or Applied Sciences High-Capacity cDNA Reverse Transcriptase Kit (43-688-13). cDNA was amplified using the Bioline SensiFast NO-ROX kit (BIO-86020), according to manufacturer’s instructions. The TaqMan GAPDH probe (Mm99999915_g1) was used as a housekeeping gene for each sample. The TaqMan probes Cyp1a1 (Mm00487218_m1), Ahr (Mm00478932_m1), Rorc (Mm01261019_g1), and Il17a (Mm00439618_m1) were used to measure transcript levels from the samples. Results were analyzed with the relative quantity (ΔΔCq) method.

### CD4 T cells isolation and differentiation

Naïve CD4 T cells were harvested and skewed as previously described^52^. After skewing, cells were washed and frozen for qPCR analysis or treated with an Ahr agonist (I_3_S-250 µM, Sigma-Aldrich I3875), antagonist (CH223191-10 µM, Tocris Bioscience 301326-22-7), or vehicle control (DMSO-Fisher Scientific D128-1) for 24 h prior to freezing.

### Tissue harvest and digestion

After experimental manipulation, mice were perfused with 0.9% saline plus 5 units/mL heparin (Medefil; MIH-3333) and tissues of interest were harvested and processed for flow cytometry as described below.

### Small intestine

Whole small intestine was collected from the animals, flayed open and rinsed with ice cold HBSS -/- (Gibco, 14175-095). Tissue was cut into ~ 2 cm pieces and stored in 30 mL of 5% FBS (R&D systems, S12450H) in HBSS until processing. Small intestine was shaken at 37 °C for 20 min to remove mucus and debris. Gut pieces were filtered over mosquito net, placed in fresh 30 mL of 5% FBS in HBSS, and shaken at 37 °C for another 20 min. Samples were again filtered over mosquito net. Pieces were cut using a razor blade until fine slurry was created. Slurry was incubated in gut digestion buffer: Collagenase 8 (Sigma, C2139-5G), DNAse (Worthington, LS002139) in 5% FBS in HBSS for 40 min, shaken at 150 rpm at 37 °C. Once digested, the solution was filtered through a 70 μm filter and washed three times with 5% FBS in HBSS.

### Lymph nodes and Peyer’s patches

A single cell suspension in RPMI was prepared from Peyer’s Patches and lymph nodes after fat removal by mechanical dissociation and subsequent filtration using sterile 70 μm filters.

### Meninges

Meninges were dissected from skull caps in ice cold RPMI and digested in the digestion buffer: Collagenase 2 (Gibco, 17101-015), collagenase D (Sigma, 11088882001) and DNAse (Worthington, LS002139) for 20 min at 37 °C. Once digested, meninges were physically dissociated with a 1 mL pipette and filtered through a sterile 70 μm filter.

### Flow cytometry

Single cell suspensions were incubated with CD16/32 Fc Block and then stained with a 1:200 antibody dilution (1:100 for transcription factors). For intranuclear staining, the eBioscience FoxP3/Transcription Factor Staining Kit (00-5523-00) was used per manufacturer’s instructions. Antibodies used were as follows: 488-conjugated CD8 (53-008182), APCe780-conjugated TCRβ (47-5961-82), e450-conjugated CD4 (48-0042-820, PE-conjugated ROR$\upgamma$t (12-6981-82), and PE-Cy7-conjugated FoxP3 (25-5773-82), all purchased from Invitrogen. APC-conjugated CD45.2 (109813) was purchased from BioLegend. A Live/Dead discrimination dye Ghost Dye Violet 510 (Tonbo Biosciences; 13-0870) was used on all samples. OneComp eBeads (Thermo Fisher Scientific, 01-111-42) were used for all color controls except for the viability dye in which cells were used. Flow cytometry was performed using a Beckman Coulter Gallios flow cytometer and data were analyzed with FlowJo software v10.7.1.

### Statistical analysis

All statistical analyses-except those associated with DeepLabCut-were performed in GraphPad Prism 9. Analyses involving two groups were performed using a two-tailed T test. If the variances between groups were significantly different, a Welch’s correction was applied. Outliers were excluded if they fell more than two standard deviations from the mean. For all analyses, the threshold for significance was at *p* < 0.05. Repeats for each experiment are specified in the figure legend corresponding to the respective panel. All p values and statistical tests are reported in Supplemental Table 1.

## Supplementary Information

Supplementary Information 1.

Supplementary Information 2.
