# Supplementary material for: Stress-induced despair behavior develops independently of the Ahr-RORγt axis in CD4 + cells
Source: Sci Rep. 2022 May 21;12:8594. doi: 10.1038/s41598-022-12464-2 (PMC9124178; doi:10.1038/s41598-022-12464-2)
Supplement: Supplementary file 2 — Supplementary Information 2. [file 41598_2022_12464_MOESM2_ESM.pdf]

Stress-Induced Despair Behavior Develops Independently of the Ahr-RORgt Axis in CD4+ cells

Courtney R. Rivet-Noor[1,2,3,$], Andrea R. Merchak[1,2,3,$], Sihan Li[2,4], Rebecca M. Beiter[1,2,3], Sangwoo Lee[5], Jalon Aaron Thomas[5], Anthony Fernández-Castañeda [1,2,3], Jung-Bum Shin[2,4] and Alban Gaultier[1,2,3,#].

[1]Center for Brain Immunology and Glia, [2]Department of Neuroscience, [3]Graduate Program in Neuroscience, [4]Graduate program in Biochemistry and Molecular Genetics, [5]Undergraduate Department of Computer Science, University of Virginia School of Medicine, Charlottesville, VA 22908, USA.

[$]C.R.N. and A.R.M. contributed equally to this work, [#]Corresponding author. Email: ag7h@virginia.edu.

Running Title: Depression Develops without Th17 Cells.

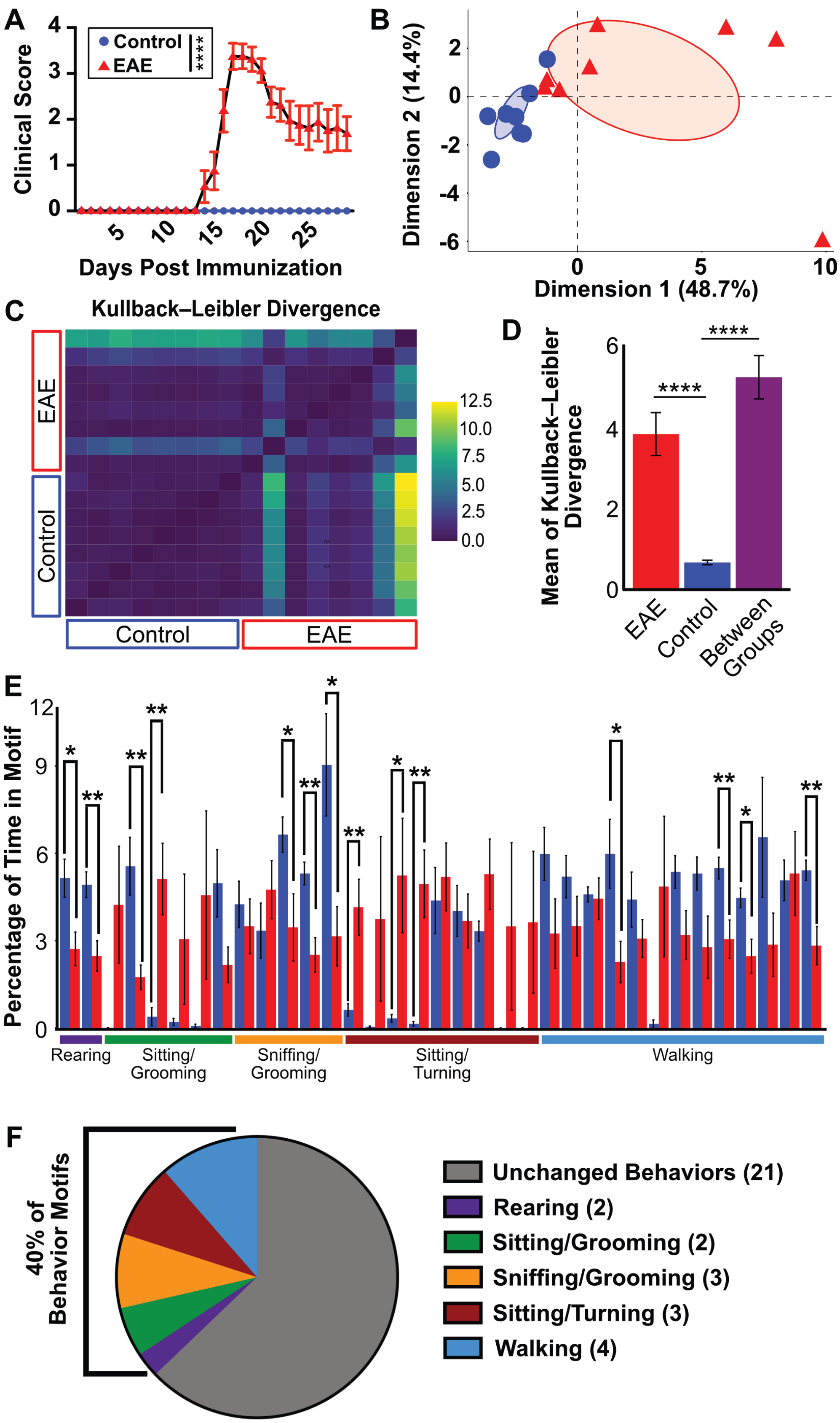
A
Control
EAE
****
Clinical Score
Days Post Immunization
B
Dimension 1 (48.7%)
Dimension 2 (14.4%)
C
Kullback–Leibler Divergence
EAE
Control
Control
EAE
D
Mean of Kullback–Leibler Divergence
****
****
EAE
Control
Between Groups
E
Percentage of Time in Motif
Rearing
Sitting/ Grooming
Sniffing/ Grooming
Sitting/ Turning
Walking
F
40% of Behavior Motifs
Unchanged Behaviors (21)
Rearing (2)
Sitting/Grooming (2)
Sniffing/Grooming (3)
Sitting/Turning (3)
Walking (4)

Supplemental Figure 1: Validation of DeepLabCut in EAE Mice. (A) Average clinical scores of EAE and control mice (n=8/group). Mann-Whitney U Test (p= <0.0001) . (B) PCA and (C) Kullback-Leibler Divergence plots representing differences between EAE and control animals (n=8/group). (D) Means of Kullback-Leibler Divergence scores in control mice, EAE mice, and between groups. Multiple T tests (p= <0.0001). (E) Percentage and grouping of motif usage by EAE vs control mice in DeepLabCut analyzed videos (n=8/group). T tests (Supplemental Table 1). (F) Quantification and grouping of significantly changed motifs (by % usage) in EAE vs control mice. N=1, All female mice.

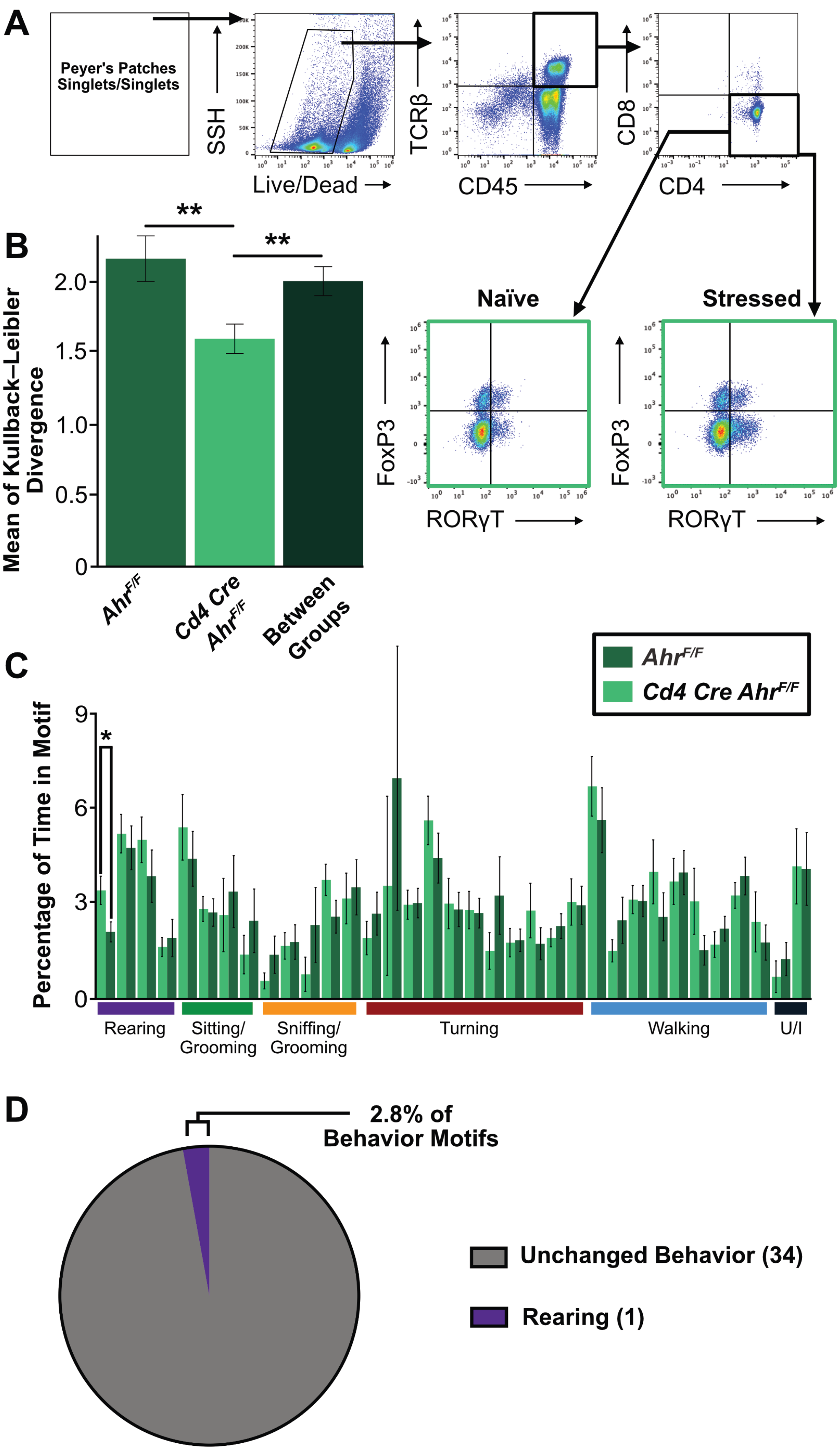

A
Peyer's Patches Singlets/Singlets
SSH
Live/Dead
TCRβ
CD45
CD8
CD4
B
Mean of Kullback–Leibler Divergence
**
**
Ahr F/F
Cd4 Cre Ahr F/F
Between Groups
Naïve
Stressed
FoxP3
RORγT
C
Percentage of Time in Motif
Ahr F/F
Cd4 Cre Ahr F/F
*
Rearing
Sitting/ Grooming
Sniffing/ Grooming
Turning
Walking
U/I
D
2.8% of Behavior Motifs
Unchanged Behavior (34)
Rearing (1)

Supplemental Figure 2: *Ahr* Depletion does not Impact Subtle Behaviors After Stress Exposure. (A) Representative flow cytometry gating strategy for Th17s in naïve vs stressed mice. (B) Graphical quantification of mean of Kullback-Leibler Divergence scores in *Ahr* KO, littermate controls, and between groups. Multiple T tests ($p = 0.0034$ and $p = 0.0056$). (C) Percentage and grouping of motif usage from DeepLabCut analyzed behaviors between *Ahr* KO and littermate controls (n= 13-14/group). T tests (Supplemental Table 1). (D) Percentage and grouping of significantly changed motifs (by % usage) between *Ahr* KO and control groups.

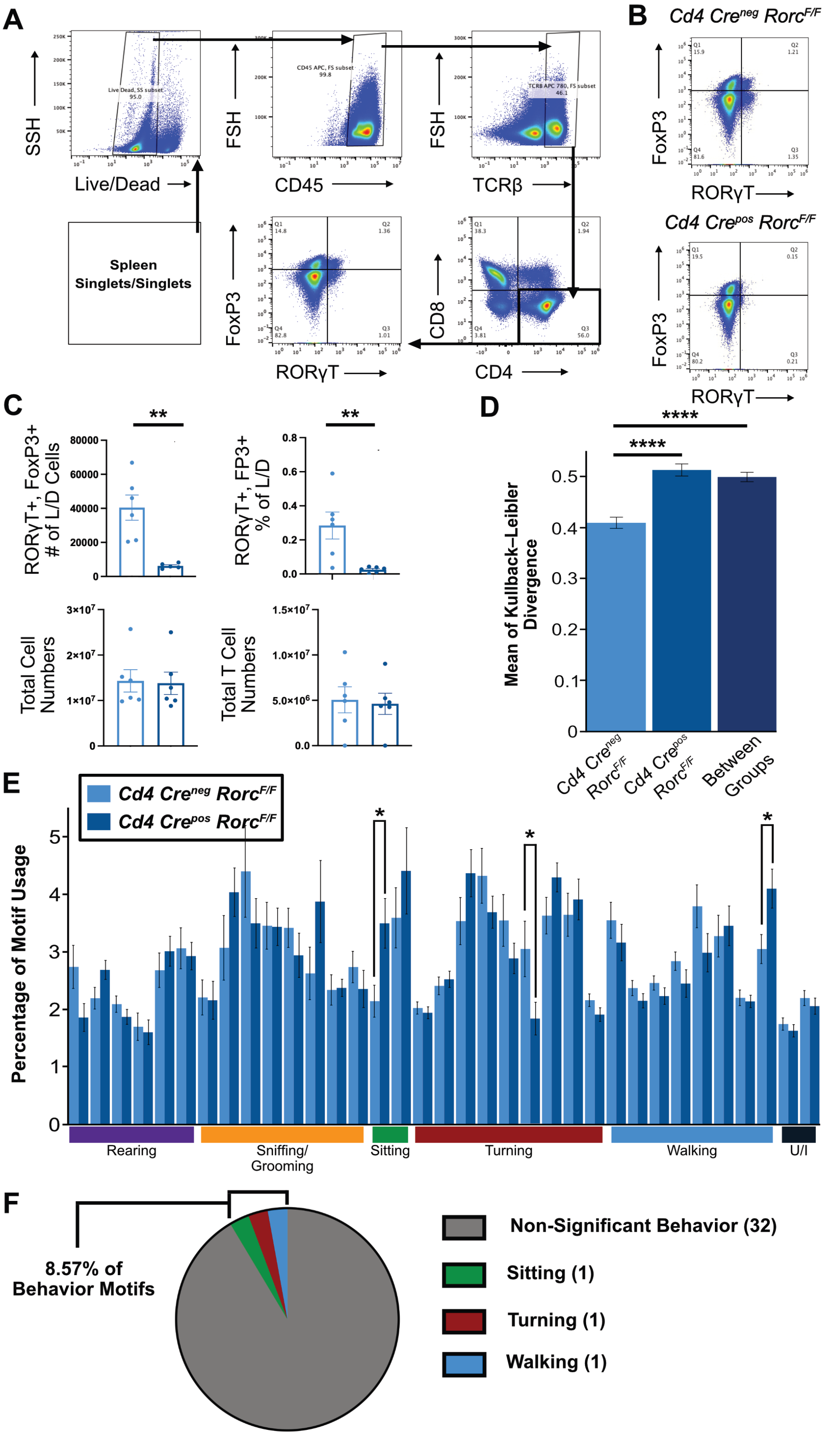

A
SSH
Live/Dead
FSH
CD45
FSH
TCRβ
Spleen Singlets/Singlets
FoxP3
RORγT
CD8
CD4
B
Cd4 Cre^neg Rorc^F/F
FoxP3
RORγT
Cd4 Cre^pos Rorc^F/F
FoxP3
RORγT
C
RORγT+, FoxP3+ # of L/D Cells
RORγT+, FP3+ % of L/D
Total Cell Numbers
Total T Cell Numbers
D
Mean of Kullback–Leibler Divergence
Cd4 Cre^neg Rorc^F/F
Cd4 Cre^pos Rorc^F/F
Between Groups
E
Cd4 Cre^neg Rorc^F/F
Cd4 Cre^pos Rorc^F/F
Percentage of Motif Usage
Rearing
Sniffing/ Grooming
Sitting
Turning
Walking
U/I
F
8.57% of Behavior Motifs
Non-Significant Behavior (32)
Sitting (1)
Turning (1)
Walking (1)

Supplemental Figure 3: *Rorc* Depletion does not Impact Subtle Behaviors in Male Mice at Baseline. (A) Representative gating strategy for spleen Foxp3 and RORγT positive cells between *Rorc* KO and control animals. (B) Representative flow cytometry quadrants for FoxP3 and RORγT+ cells between *Rorc* KO and littermate controls. (C) Quantification of the number and percent of mesenteric RORγT+ and FoxP3+ cells and total cell and T cell numbers between *Rorc* KO and littermate controls (n=5-6/group). T tests (Number of RORgT, FP3+ cells: p= 0.0048, RORgt, FoxP3+ % of L/D: p= 0.0085). (D) Means of Kullback-Leibler Divergence scores in littermate controls, *Rorc* KO mice, and between groups (n=22 or 32/group). Multiple T tests (p= <0.0001). (E) Percentage and grouping of motif usage by *Rorc* KO vs littermate controls in DeepLabCut analyzed videos (n=22 or 32/group). T tests (Supplemental Table 1). (F) Quantification and grouping of significantly changed motifs (by % usage) in *Rorc* KO vs littermate controls. Male mice.

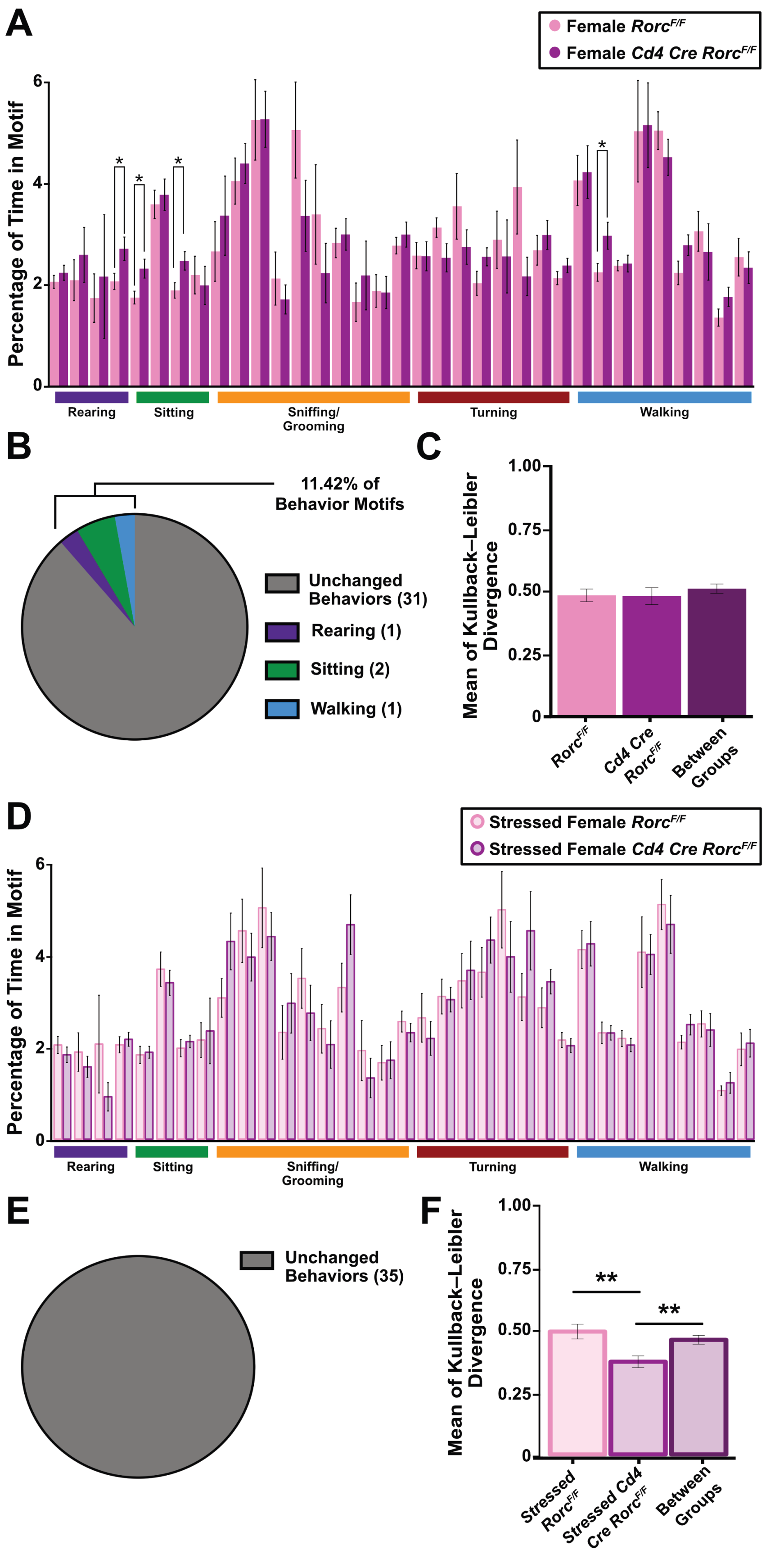
A
Female $Rorc^{F/F}$
Female $Cd4$ Cre $Rorc^{F/F}$
Percentage of Time in Motif
Rearing
Sitting
Sniffing/ Grooming
Turning
Walking
B
11.42% of Behavior Motifs
Unchanged Behaviors (31)
Rearing (1)
Sitting (2)
Walking (1)
C
Mean of Kullback–Leibler Divergence
$Rorc^{F/F}$
$Cd4$ Cre $Rorc^{F/F}$
Between Groups
D
Stressed Female $Rorc^{F/F}$
Stressed Female $Cd4$ Cre $Rorc^{F/F}$
Percentage of Time in Motif
Rearing
Sitting
Sniffing/ Grooming
Turning
Walking
E
Unchanged Behaviors (35)
F
Mean of Kullback–Leibler Divergence
Stressed $Rorc^{F/F}$
Stressed $Cd4$ Cre $Rorc^{F/F}$
Between Groups

Supplemental Figure 4: ***Rorc* Depletion does not Impact Subtle Behaviors in Female Mice at Baseline or after Stress.** (A) Percentage and grouping of baseline motif usage by female *Rorc* KO vs littermate controls in DeepLabCut analyzed videos (n=12/group). T tests (Supplemental Table 1). (B) Means of baseline Kullback-Leibler Divergence scores in female littermate controls, *Rorc* KO mice, and between groups (n=12/group). Multiple T tests (Supplemental Table 1). (C) Quantification and grouping of significantly changed motifs (by % usage) at baseline in female *Rorc* KO vs littermate controls. (D) Percentage and grouping of motif usage by stressed female *Rorc* KO vs stressed female littermate controls after 3 weeks of UCRS in DeepLabCut analyzed videos (n=12/group). T tests (Supplemental Table c1). (E) Quantification and grouping of significantly changed motifs (by % usage) after 3 weeks of UCRS in female *Rorc* KO vs littermate controls. (F) Means of Kullback-Leibler Divergence scores after 3 weeks of UCRS in female littermate controls, *Rorc* KO mice, and between groups (n=12/group). Multiple T tests (p= 0.0015, p= 0.0032).

Day	UCS Stress Procedure
1	Restraint + Cage Tilt
2	Cage Crowding + 2x Cage Change
3	Strobe Light + Wet Bedding
4	White Noise + 24hr Light Exposure
5	Restraint + 2x Cage Change
6	Cage Crowding + Wet Bedding
7	Strobe Light + Cage Tilt

Supplemental Table 2: Unpredictable Chronic Stress Protocol Table

Day	UCRS Stress Procedure
1	Restraint + Cage Tilt
2	Restraint + 2x Cage Change
3	Restraint + Wet Bedding
4	Restraint + Cage Tilt
5	Restraint + 2x Cage Change
6	Restraint + Wet Bedding
7	Restraint + Cage Tilt

Supplemental Table 3: Unpredictable Chronic Restraint Stress Protocol Table.
